# Coordinated Transcriptional Repression of CAV1 and CAV2 in Thoracic Aortic Aneurysm: A microRNA Regulatory Network Analysis

**DOI:** 10.3390/genes17070827

**Published:** 2026-07-20

**Authors:** Dimitrios E. Magouliotis, Serge Sicouri, Vasiliki Androutsopoulou, Massimo Baudo, Thanos Athanasiou, Dimitrios V. Avgerinos, John Skoularigis, Grigorios Giamouzis, Basel Ramlawi, Andrew Xanthopoulos

**Affiliations:** 1 Department of Cardiac Surgery Research, Lankenau Institute for Medical Research, Wynnewood, PA 19096, USA; magouliotisd@mlhs.org (D.E.M.); sicouris@mlhs.org (S.S.); massimo.baudo@icloud.com (M.B.); ramlawib@mlhs.org (B.R.); 2 Department of Cardiothoracic Surgery, Faculty of Medicine, University of Thessaly, 41110 Larissa, Greece; androutsopoulouvasiliki@uth.gr; 3 Department of Surgery and Cancer, Imperial College London, London W12 0NN, UK; t.athanasiou@imperial.ac.uk; 4 Department of Cardiac Surgery, Onassis Cardiac Surgery Center, 17674 Athens, Greece; davgerinos@gmail.com; 5 Department of Cardiology, University Hospital of Larissa, Biopolis, 41110 Larissa, Greece; iskoular@gmail.com (J.S.); grgiamouzis@gmail.com (G.G.); 6 Department of Cardiac Surgery, Lankenau Heart Institute, Main Line Health, Wynnewood, PA 19096, USA

**Keywords:** thoracic aortic aneurysm, CAV1, CAV2, caveolin, transcriptomics, microRNA, gene regulation, endothelial dysfunction, differential gene expression

## Abstract

**Background:** Thoracic aortic aneurysm (TAA) is a potentially life-threatening degenerative disease whose principal danger arises from progressive aortic dilation with the attendant risk of rupture and dissection and which is characterized by extracellular matrix breakdown, smooth muscle loss, and endothelial dysfunction. Caveolae, plasma membrane microdomains built from caveolins (CAV1-3) and cavins (CAVIN1-4), govern nitric oxide (NO) signaling, endocytosis, and mechanotransduction. We hypothesized that downregulation of caveolae-associated genes, driven in part by microRNAs, contributes to endothelial failure and vascular remodeling in TAA. **Methods:** Normalized transcriptomic expression values for five caveolae-associated genes were retrieved from the GSE26155 dataset (43 TAA and 43 control aortas) using GEO2R. Differential expression was assessed for CAV1, CAV2, CAV3, CAVIN1, and CAVIN2, and Spearman correlation with Deming regression explored inter-gene relationships. Functional enrichment (Enrichr) and experimentally validated microRNA-target interactions (miRTarBase) were used to infer regulatory and mechanistic networks. CpG island mapping and gene-gene interactome construction (GeneMANIA) complemented the analyses. **Results:** CAV1 and CAV2 were downregulated in TAA at nominal significance (CAV1, *p* = 0.0225; CAV2, *p* = 0.0361); after Benjamini–Hochberg correction across the five candidate genes both differences attenuated to a consistent trend (*q* approximately 0.09), while the two caveolins were strongly co-expressed (Spearman *r* = 0.527, *p* < 0.001; Deming CAV2 = 1.881 × CAV1-0.892), indicating coordinated transcriptional regulation. Network analysis linked both genes to NOS3, NOSTRIN, EGFR, HRAS, and RAC1, consistent with impaired endothelial nitric oxide and GTPase signaling. Gene Ontology enrichment highlighted endothelial proliferation, nitric oxide metabolism, calcium homeostasis, vesicle organization, and MAPK regulation. Database-supported analysis (miRTarBase) identified miR-93-5p, miR-199a-3p, miR-203a-3p, and the miR-29 family as experimentally validated candidate repressors of CAV1/CAV2. **Conclusions:** This integrative transcriptomic and microRNA analysis identifies coordinated CAV1 and CAV2 downregulation as a candidate molecular event in thoracic aortic aneurysm, associated with caveolar loss, endothelial dysfunction, and disrupted nitric oxide homeostasis. The CAV1/CAV2-microRNA axis represents a candidate mechanistic signature warranting further investigation as a potential therapeutic target in aortic disease.

## 1. Introduction

Thoracic aortic aneurysm (TAA) is a progressive and potentially lethal condition defined by pathological dilation of the aortic wall, driven by medial smooth muscle cell attrition, elastic fiber fragmentation, and extracellular matrix remodeling [1]. Although surgical repair has advanced considerably, the molecular determinants of wall weakening and rupture remain incompletely characterized. Current paradigms foreground proteolytic degradation, maladaptive inflammation, and oxidative injury, yet accumulating evidence positions endothelial dysfunction and aberrant mechanotransduction as initiating rather than secondary events in aneurysm biology [1,2,3].

The vascular endothelium integrates pulsatile hemodynamic forces into molecular signals that govern nitric oxide (NO) bioavailability, redox homeostasis, and vascular tone. Endothelial compromise leads to eNOS uncoupling, reactive oxygen species accumulation, and sustained low-grade inflammation, each of which is well-documented in aneurysmal tissue [4,5,6,7]. At the structural interface between the plasma membrane and these signaling cascades lie caveolae: 50–100 nm flask-shaped microdomains enriched in the sphingolipid-cholesterol phase that serve as mechanosensors, endocytic portals, and signaling platforms [6,7,8,9]. Caveolae are scaffolded by three caveolins (CAV1, CAV2, CAV3) and four cavins (CAVIN1-CAVIN4), whose stoichiometric assembly is required for caveolar biogenesis and stability.

Among caveolins, CAV1 and CAV2 are the dominant isoforms in vascular endothelial and smooth muscle cells. CAV1 anchors endothelial NO synthase (eNOS, encoded by NOS3) within caveolar membranes, tethering it to essential cofactors including calmodulin and tetrahydrobiopterin; this interaction is essential for maintaining eNOS in a coupled, NO-producing state [6,7,8,9]. Disruption of CAV1 function releases eNOS from scaffolding constraints, promoting uncoupled superoxide generation and depleted NO bioavailability, a molecular shift that accelerates endothelial dysfunction and oxidative tissue injury [6,8]. Beyond eNOS regulation, caveolins coordinate receptor-mediated endocytosis, cholesterol trafficking, small GTPase signaling, and calcium flux, intersecting multiple pathways implicated in TAA pathobiology [6,7,8,9]. Single-cell and spatial transcriptomic studies of human aortic tissue have highlighted endothelial programs related to calcium handling, cytoskeletal remodeling, and mechanostress response, processes directly coupled to caveolar function [5,6,7].

Prior work from our group has characterized transcriptomic dysregulation in TAA, focusing on gap-junction proteins, cadherin-5, tight-junction molecules, and epigenetic regulatory signatures [4,8,9,10]. The present investigation extends this program to the caveolin axis, a molecular system whose specific contribution to human TAA remains uncharacterized at the transcriptomic level. Rather than reapplying a generic bioinformatics pipeline, this study is designed around a focused mechanistic hypothesis: that coordinated repression of CAV1 and CAV2, mediated in part by microRNAs, constitutes a discrete molecular event linking membrane microdomain dysfunction to endothelial failure and vascular remodeling in TAA. We test this hypothesis using the GSE26155 human transcriptomic dataset, combining differential expression, correlation-regression analysis, functional enrichment, protein interaction networking, and miRNA target mapping to delineate the caveolin-centered regulatory circuit in aneurysmal disease.

## 2. Materials and Methods

### 2.1. Hypothesis and Study Design

**Figure 1:**
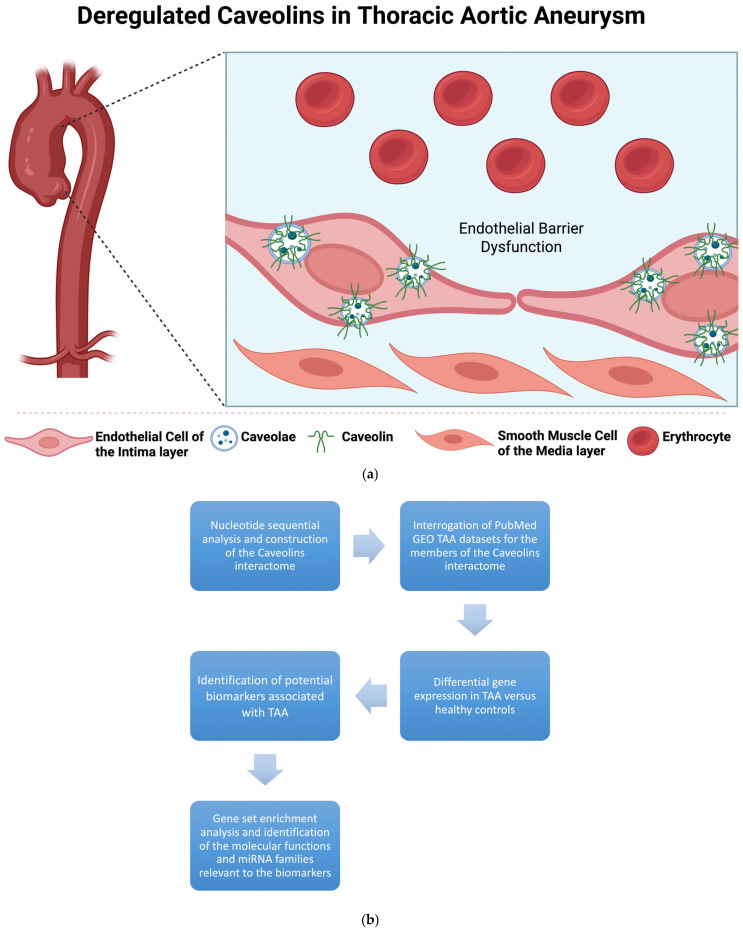
(**a**) Hypothesis illustrating the role of caveolin dysregulation in thoracic aortic aneurysm (TAA), depicting endothelial barrier dysfunction associated with deregulated caveolins in the intima and smooth muscle cells of the media layer. (**b**) Flowchart demonstrating the analytical steps of the study, from nucleotide sequence analysis and interactome construction through GEO dataset interrogation, differential gene expression analysis, biomarker identification, and gene set enrichment analysis.

We hypothesized that transcriptional dysregulation of caveolae-associated genes, particularly CAV1 and CAV2, contributes to TAA through impaired endothelial signaling and mechanotransduction (Figure 1a). Specifically, we posited that downregulation of CAV1-3 and their post-transcriptional inhibition by selected microRNAs leads to eNOS uncoupling, altered calcium homeostasis, and endothelial dysfunction, as supported by prior experimental models of caveolin deficiency and vascular remodeling [6,8,11]. This study adhered to the STREGA (STrengthening the REporting of Genetic Association Studies) recommendations [12]. A bioinformatics-driven framework was used to interrogate publicly available transcriptomic datasets and identify potential biomarkers and therapeutic targets in TAA. Central to this approach was the construction of gene-gene interaction networks to delineate the interactome of differentially expressed genes (DEGs) and their participation in key pathogenic pathways.

### 2.2. Transcriptomic Dataset and Gene Extraction

Publicly available human transcriptomic data were queried through the Gene Expression Omnibus (GEO) at the National Center for Biotechnology Information (https://www.ncbi.nlm.nih.gov/gds; accessed 20 March 2025). Two researchers (D.E.M., S.S.) independently searched GEO using the terms “thoracic aortic aneurysm”, “TAA”, and “aortic dilatation”, restricted to Homo sapiens. Reviewer agreement was quantified using the kappa coefficient. Dataset GSE26155 was selected following systematic screening as previously detailed [13]. The dataset comprises 86 samples (43 TAA and 43 non-aneurysmal control thoracic aortas) generated on the Affymetrix Human Exon 1.0 ST Array, a microarray platform; no RNA-sequencing data were used in this study. TAA samples were obtained from dilated aortic segments (>45 mm); controls were non-dilated (<40 mm) tissues from deceased donors (mean diameter 34.1 plus or minus 3.6 mm versus 53.6 plus or minus 7.5 mm in TAA). All patients had tricuspid aortic valves; 59% had aortic stenosis and 53% had regurgitation. No samples with a lumen diameter between 40 and 45 mm were included. Normalized, log2-scaled expression values for five caveolae-associated genes (CAV1, CAV2, CAV3, CAVIN1, CAVIN2) were retrieved using GEO2R (https://www.ncbi.nlm.nih.gov/geo/geo2r/; accessed 20 March 2025), the NCBI tool that applies the GEOquery (v2.74.0) and limma (v3.54.0) R packages to the normalized series matrix deposited for the dataset. Each gene was represented by a single transcript-cluster probe set on the platform, and the corresponding per-sample values were therefore used directly, so no summarization across multiple probes mapping to the same gene was required; complete values were available for all 86 samples. These per-sample values were exported to GraphPad Prism for statistical analysis.

### 2.3. Statistical Analysis and Regression Modeling

Correlations among caveolae-associated genes were examined using Spearman’s rank correlation. Deming regression was applied to model the quantitative relationship between CAV1 and CAV2, reflecting mutual biological dependence rather than one-directional prediction. Receiver Operating Characteristic (ROC) curves and corresponding Area Under the Curve (AUC) values were computed to estimate the discriminatory capacity of individual caveolins for TAA versus control status. Between-group comparisons used two-tailed unpaired *t*-tests with Welch’s correction for approximately normally distributed data and Mann–Whitney U tests for non-parametric distributions, with statistical significance defined as two-sided *p* < 0.05 [14]. Because a focused panel of five a priori candidate genes was examined, raw *p*-values are reported together with Benjamini–Hochberg adjusted values (*q*) to account for multiple comparisons. Internal validation was performed using Principal Component Analysis (PCA) on caveolin expression values to assess sample clustering. Expression values were obtained through GEO2R, which executes the GEOquery and limma R packages; all downstream statistical analyses and plots were generated in GraphPad Prism (version 10.0; GraphPad Software, San Diego, CA, USA). Selected schematic figures were created using BioRender (https://BioRender.com).

### 2.4. Protein Interaction Network Construction

Protein-protein and functional interaction networks were generated using GeneMANIA (https://genemania.org) [15]. This platform integrates co-expression, physical interaction, pathway co-annotation, and shared protein domain data to construct biologically meaningful interaction maps. Nodes representing interactors with established roles in endothelial function, including NOS3, NOSTRIN, EGFR, HRAS, and RAC1, were highlighted in the analysis [6,7,8,11]. Node size and edge thickness were scaled according to interaction strength and co-expression probability.

### 2.5. Gene Set Enrichment and microRNA Analysis

Gene Ontology (GO) enrichment analysis was performed using Enrichr (https://maayanlab.cloud/Enrichr; 21 March 2025) [16]. The “GO Biological Process 2025” and “GO Molecular Function 2025” libraries were queried to identify overrepresented pathways among DEGs. Adjusted *p*-values were computed via the Benjamini–Hochberg correction, with the significance threshold set at adjusted *p* < 0.01. Predicted and validated microRNA regulators of CAV1 and CAV2 were retrieved from miRTarBase (version 2023), restricted to experimentally validated interactions [17]. Candidate miRNAs were prioritized based on target overlap and prior associations with endothelial remodeling or aortic disease. CpG island mapping of CAV1 and CAV2 promoter loci was performed using EMBOSS CpGPlot (v6.6.0.0) to identify potential regions of epigenetic modulation [18].

## 3. Results

### 3.1. Differential Expression of Caveolins and Cavins in TAA

**Figure 2:**
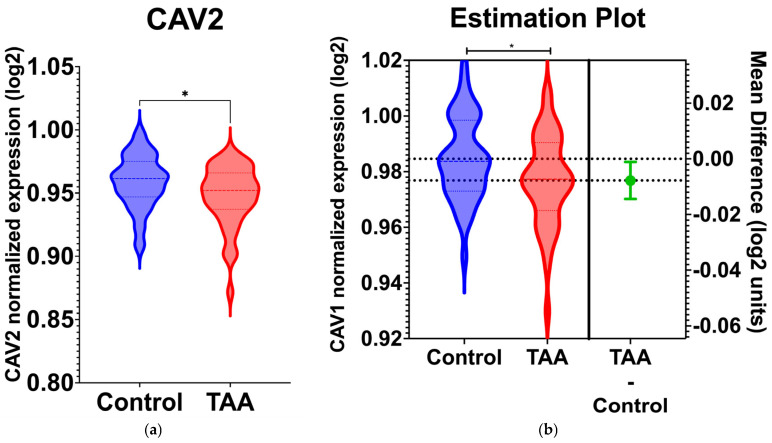
(**a**) Violin plot of CAV2 normalized (log2) expression in normal control and dilated (TAA) thoracic aortic tissue samples; CAV2 was downregulated in dilated aortic tissue compared with control (Mann–Whitney U test, *p* = 0.0361). (**b**) Gardner–Altman estimation plot of CAV1 normalized (log2) expression between control and TAA groups, with the mean difference and its 95% confidence interval shown on the right axis; CAV1 was downregulated in TAA (two-tailed unpaired *t*-test with Welch’s correction, *p* = 0.0225). Both differences were nominally significant and attenuated to a consistent trend after the Benjamini–Hochberg correction across the five candidate genes (*q* approximately 0.09). *: *p* < 0.005.

The analytical workflow is illustrated in Figure 1b, and the STREGA checklist is provided in File S1. Following retrieval of normalized expression values from the GSE26155 dataset, five caveolae-associated genes (CAV1, CAV2, CAV3, CAVIN1, CAVIN2) were analyzed. A comparison between TAA and control samples showed downregulation of CAV1 and CAV2 in aneurysmal tissue at nominal significance (CAV1, two-tailed unpaired *t*-test with Welch’s correction, *t* = 2.325, df = 83.43, *p* = 0.0225; CAV2, Mann–Whitney U test, *p* = 0.0361), whereas CAV3, CAVIN1, and CAVIN2 showed no significant between-group difference (all *p* > 0.05). After the Benjamini–Hochberg correction across the five candidate genes, the CAV1 and CAV2 differences attenuated to a consistent downward trend (*q* approximately 0.09 for both), and the effect sizes were modest. Accordingly, the primary evidence for coordinated caveolin repression derives from the strong CAV1-CAV2 co-expression relationship described below rather than from the individual between-group thresholds. The absence of significant changes in CAV3, CAVIN1, and CAVIN2 is itself informative: it is consistent with an isoform-specific rather than a global caveolar transcriptional change, in keeping with the established predominance of the CAV1-CAV2 heterodimer in large-vessel endothelial biology and with prior data showing that CAV3 is largely restricted to cardiomyocytes and skeletal muscle. Violin and estimation plots (Figure 2) depict the distribution of normalized expression values and the magnitude of the difference in TAA relative to controls.

### 3.2. CAV1-CAV2 Correlation and Regression Analysis

**Figure 3:**
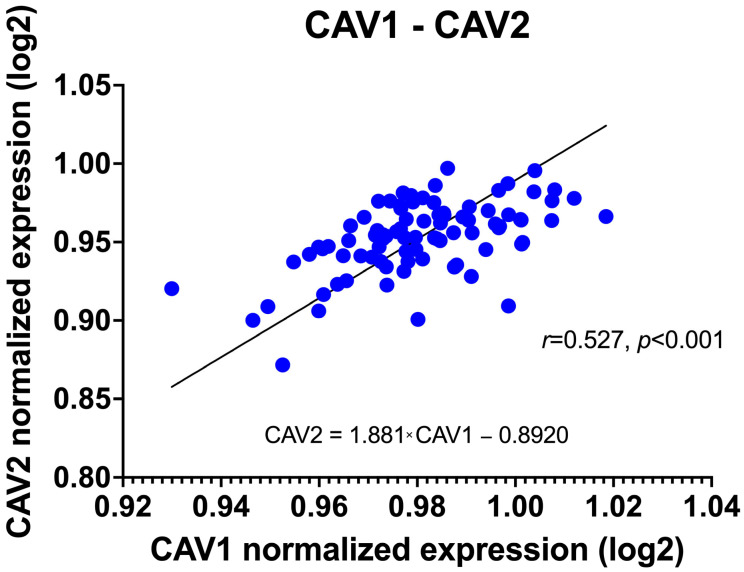
Scatterplot of CAV1 versus CAV2 normalized (log2) expression across the GSE26155 cohort (abscissa, CAV1 normalized expression; ordinate, CAV2 normalized expression). A strong positive association was identified by Spearman’s rank correlation (r = 0.527, 95% CI 0.349 to 0.668, *p* < 0.001; *n* = 86). Deming regression yielded the equation CAV2 = 1.881 × CAV1 − 0.892, consistent with coordinated transcriptional co-regulation of the two caveolins in both control and aneurysmal aortic tissue.

Spearman’s rank correlation identified a strong positive relationship between CAV1 and CAV2 expression across the cohort (*r* = 0.527, 95% CI 0.349 to 0.668, *p* < 0.001; *n* = 86 pairs), indicating coordinated transcriptional regulation (Figure 3). This co-expression relationship is independent of the between-group significance thresholds and is robust to multiple-comparison correction, and it therefore provides the principal evidence that CAV1 and CAV2 are co-regulated. Deming regression produced the equation CAV2 = 1.881 × CAV1 − 0.892 (slope 95% CI 1.157 to 2.606, *p* < 0.001), consistent with proportional co-variation in the two genes. ROC analysis demonstrated only fair discriminative capacity for CAV1 and CAV2 individually in distinguishing TAA from control tissue (AUC approximately 0.63 for each). These values reflect the modest diagnostic yield expected of individual transcriptomic markers derived from a single dataset; CAV1 and CAV2 are best understood as components of a broader regulatory signature rather than standalone biomarkers, and any clinical applicability would require multi-marker panels and prospective validation in independent cohorts.

### 3.3. Protein–Protein Interaction Network

**Figure 4:**
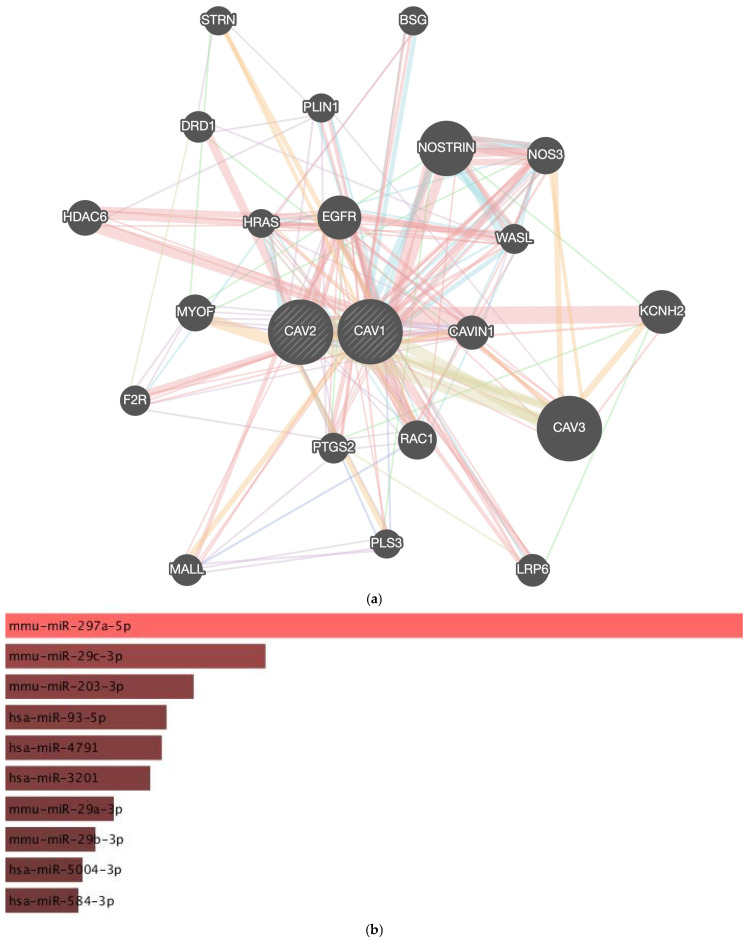
(**a**) Protein–protein interaction network of CAV1 and CAV2 generated by GeneMANIA, highlighting functional associations with NOS3, NOSTRIN, EGFR, HRAS, CAV3, CAVIN1, and RAC1. Edge colors represent different interaction evidence types (co-expression, physical interaction, pathway co-annotation). Node size is proportional to interaction degree. (**b**) Bar graph of experimentally validated microRNAs targeting CAV1 and CAV2 according to miRTarBase, ranked by evidence score. The miR-29 family, miR-93-5p, and miR-203-3p represent the most prominent experimentally validated regulators.

Network analysis through GeneMANIA web server (https://genemania.org/; accessed 20 March 2025) identified a dense functional cluster centered on CAV1 and CAV2 (Figure 4a). High-confidence edges linked both genes with endothelial nitric oxide synthase (NOS3), nitric oxide synthase trafficking regulator (NOSTRIN), epidermal growth factor receptor (EGFR), HRas proto-oncogene GTPase (HRAS), and Ras-related C3 botulinum toxin substrate 1 (RAC1). Co-expression and pathway connectivity patterns suggest that loss of caveolar components may simultaneously disrupt nitric oxide signaling, vesicle-mediated transport, and GTPase-dependent cytoskeletal remodeling. Secondary network associations with DNM2 and ACTB further reinforced a mechanistic link between caveolar disassembly and actin-cytoskeleton dynamics.

### 3.4. Gene Ontology Enrichment and microRNA Regulators

Enrichr-based GO analysis yielded 134 significantly enriched biological processes (adjusted *p* < 0.01). The top enriched processes included regulation of endothelial and epithelial cell proliferation, nitric oxide metabolic process, receptor-mediated endocytosis, calcium-ion homeostasis, vesicle organization, and regulation of the MAPK cascade. Additional enriched categories encompassed lipid transport, cell-junction assembly, and apoptotic signaling, collectively implicating caveolar signaling in vascular homeostasis and structural stability. Twenty-eight molecular functions reached statistical significance (adjusted *p* < 0.01), with the most enriched terms being kinase binding (GO:0019900), protein kinase binding (GO:0019901), nitric oxide synthase binding (GO:0050998), cholesterol binding (GO:0015485), and small GTPase binding (GO:0031267). Additional enrichments covered sterol binding, ATPase binding, and peptidase activator activity, consistent with a multifunctional regulatory role for CAV1 in endothelial signaling and lipid trafficking.

CpG analysis identified promoter-proximal islands within the 5′ regions of CAV1 and CAV2. These findings are hypothesis-generating rather than definitive: they identify candidate loci of epigenetic regulation whose functional significance would require bisulfite sequencing or methylation array profiling in independent aortic tissue cohorts to determine whether promoter hypermethylation contributes to the transcriptional repression observed in TAA. Analysis of experimentally validated interactions cataloged in miRTarBase identified several database-supported candidate microRNA regulators shared by both transcripts, notably miR-93-5p, miR-199a-3p, miR-203a-3p, and members of the miR-29 family (Figure 4b). These are candidate regulators only: microRNA abundance was not measured in this study, as GSE26155 contains no microRNA expression data, so neither microRNA-target expression correlation nor methylation validation was possible, and tissue-level quantification is required to confirm a regulatory role. The listed microRNAs are nonetheless established contributors to endothelial injury and extracellular matrix remodeling in vascular disease contexts.

## 4. Discussion

**Figure 5:**
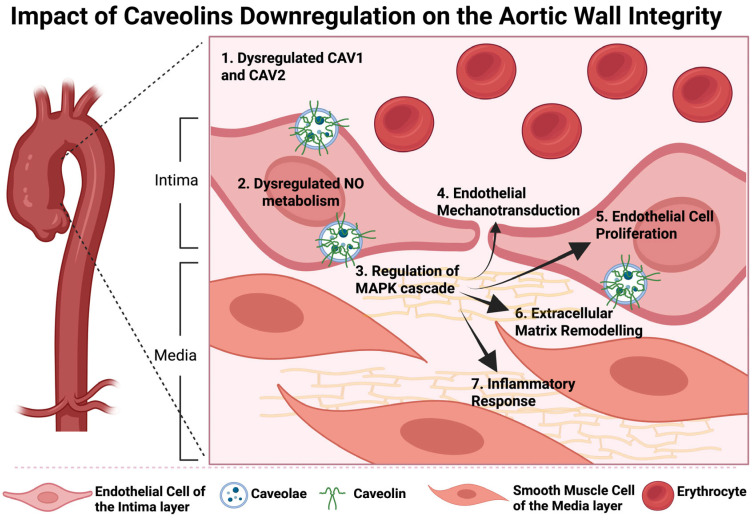
Impact of caveolin downregulation on aortic wall integrity in thoracic aortic aneurysm (TAA). Schematic summarizing downstream consequences of CAV1 and CAV2 repression on vascular homeostasis. Loss of caveolar integrity in endothelial cells of the intima leads to: (1) dysregulated CAV1 and CAV2 expression, triggering (2) impaired nitric oxide (NO) metabolism, (3) altered MAPK signaling cascade, and (4) disrupted endothelial mechanotransduction. These perturbations promote (5) abnormal endothelial cell proliferation, (6) extracellular matrix remodeling, and (7) inflammatory pathway activation in the media layer, collectively driving medial degeneration and weakening of the aortic wall.

This study provides transcriptomic evidence that the caveolae-associated genes CAV1 and CAV2 show coordinated lower expression in human thoracic aortic aneurysm tissue, an interpretation supported most strongly by their pronounced co-expression rather than by the individual between-group comparisons. This association, together with shared experimentally validated microRNA regulators, is compatible with a common upstream mechanism governing caveolar biogenesis and endothelial homeostasis, although the present cross-sectional design demonstrates association and cannot establish causality (Figure 5). Functional enrichment analysis converged on biological processes and molecular functions characteristic of caveolar biology: nitric oxide signaling, calcium-ion homeostasis, vesicle organization, receptor-mediated endocytosis, and lipid trafficking [6,7,8,9,19,20,21,22,23,24,25,26,27]. The identification of miR-93-5p, miR-199a-3p, miR-203a-3p, and the miR-29 family as experimentally validated candidate repressors of CAV1/2 provides a plausible basis for post-transcriptional silencing that may contribute to endothelial dysfunction under aneurysmal stress conditions [27,28,29].

Caveolins perform indispensable functions in vascular endothelium. CAV1 provides structural scaffolding that anchors eNOS to caveolar membranes, maintaining coupling to cofactors such as calmodulin and tetrahydrobiopterin [20,30]. Displacement of eNOS from this scaffold by CAV1 loss releases the enzyme into an uncoupled state, generating superoxide rather than NO and driving oxidative injury [26,30]. The observed co-repression of CAV1 and CAV2 in TAA may therefore represent a molecular pivot that converts the endothelial phenotype from homeostatic to maladaptive, predisposing to medial degeneration and progressive wall weakening [4,5,6,7,8,21,22,23]. Experimental corroboration comes from caveolin-1 knockout models, which exhibit loss of caveolae, endothelial hyperactivation, impaired vascular relaxation, and in some contexts, pulmonary hypertension and aortic dilation [21,22]. Genetic manipulation of CAV1 in aneurysm-prone murine models produces sex-dependent endothelial and smooth muscle abnormalities that closely parallel the human disease phenotype [11].

The interactome linking CAV1 and CAV2 with NOS3, NOSTRIN, EGFR, HRAS, and RAC1 expands the mechanistic scope of caveolin loss beyond NO metabolism. EGFR and HRAS are canonical activators of the MAPK cascade, the dysregulation of which is enriched in our GO analysis and has been independently implicated in aortic remodeling [23,24]. RAC1, a small GTPase, controls cytoskeletal dynamics and endothelial barrier function through actin polymerization; its interaction with CAV1 is required for caveolar internalization and membrane tension buffering [25,31]. Loss of caveolar scaffolding therefore plausibly disrupts not only biochemical but also mechanobiological signaling in the aneurysmal aortic wall. Secondary network nodes including DNM2 and ACTB further support a model in which caveolar disassembly compromises vesicle formation, actin dynamics, and endocytic recycling, each of which contributes to endothelial barrier integrity.

Beyond NO metabolism, the enrichment of cholesterol binding, sterol binding, and lipid transport functions in our dataset reflects caveolins’ central role in plasma membrane lipid organization [24,32,33,34]. Perturbation of sterol-enriched microdomains destabilizes receptor localization and recycling, impairing endothelial adaptability to hemodynamic stress and potentially promoting smooth muscle apoptosis and extracellular matrix degradation [2,3,6,7,8,9]. Calcium-ion homeostasis and vesicle organization, prominent in our enrichment results, align with established data showing that caveolae act as calcium entry portals and vesicular trafficking hubs in endothelial cells [25,31]. Their disruption in TAA would therefore impair multiple layers of endothelial adaptation simultaneously.

At the post-transcriptional level, this study highlights microRNA-mediated repression as a plausible, database-derived candidate contributor to caveolin silencing. miR-93-5p and miR-199a-3p have documented roles in endothelial injury and angiogenic suppression; miR-203a-3p modulates inflammatory signaling; and the miR-29 family is a well-established regulator of extracellular matrix turnover and aortic aneurysm progression [12,27,28,29,35,36]. Their convergence on CAV1/2, based on experimentally validated interactions, is compatible with a post-transcriptional circuit in which hemodynamic, inflammatory, or proteotoxic stimuli induce microRNA expression that further represses caveolin translation, amplifying an initial transcriptional deficit; this circuit remains to be tested by direct measurement of microRNA abundance in aortic tissue. Promoter-proximal CpG islands identified in CAV1 and CAV2 loci raise the additional possibility of epigenetic silencing through methylation, consistent with prior evidence linking promoter hypermethylation to aortopathy [10,37,38]. Caveolin repression in TAA may therefore reflect a convergent regulatory landscape encompassing transcriptional, epigenetic, and microRNA-mediated mechanisms.

From a clinical perspective, the caveolin axis may offer translational opportunities that warrant further study. Reduced CAV1 and CAV2 expression could, if confirmed at the protein level, serve as tissue markers of endothelial mechanodysfunction, complementing established molecular markers such as junctional proteins (CDH5, ZO-2) and connexins, which are also dysregulated in TAA [4,8,10]. Therapeutically, restoration of caveolar integrity or recoupling of eNOS activity might mitigate endothelial stress at early disease stages; antagomirs targeting miR-93-5p or the miR-29 family could in principle derepress caveolin synthesis, while interventions normalizing membrane sterol composition might stabilize caveolar architecture [27,28,29,32,36]. These therapeutic possibilities remain hypothetical: pharmacologic targeting of membrane microdomains is technically challenging, and the caveolin-microRNA axis is presented here as a candidate for future translational investigation rather than an established target.

The present work should be interpreted within its limitations. It is a hypothesis-generating computational analysis rather than an experimental study, and it demonstrates association rather than causation. First, the analysis rests on a single, cross-sectional, publicly available cohort (GSE26155); independent human thoracic aortic transcriptomic datasets with comparable phenotyping are scarce, which constrains external replication, and the findings therefore require validation in an independent cohort before generalization. Second, the between-group differences in CAV1 and CAV2 were modest in magnitude and, although nominally significant, attenuated to a consistent trend after correction for multiple comparisons across the five candidate genes; the coordinated-repression interpretation consequently rests primarily on the CAV1-CAV2 co-expression relationship, which is robust to such correction. Third, the study provides no protein-level or functional confirmation. Protein quantification of CAV1 and CAV2 by immunohistochemistry and Western blotting, direct measurement of the candidate microRNAs in aneurysmal versus control tissue, and structural assessment of caveolar density and integrity, for example, by electron microscopy or caveolin immunostaining, are each required to establish that the transcriptional signal reflects genuine caveolar loss. Fourth, the microRNA and CpG analyses are predictive and database-derived; they were not validated by measured microRNA expression, microRNA-target expression correlation, or methylation profiling. Future studies should integrate hemodynamic and clinical variables, perform bisulfite or methylation-array profiling of the CAV1 and CAV2 promoters, and conduct microRNA perturbation experiments paired with functional assessment of eNOS coupling and nitric oxide bioavailability to test mechanistic causality. Multiomic integration could further clarify the relative contributions of transcriptional, epigenetic, and post-transcriptional mechanisms to caveolar dysfunction in aneurysm formation.

## 5. Conclusions

This integrative transcriptomic and microRNA-based analysis identifies coordinated downregulation of CAV1 and CAV2 as a candidate molecular feature of thoracic aortic aneurysm, associating membrane microdomain disruption with endothelial dysfunction, nitric oxide dysregulation, and vascular remodeling. The strong inter-gene correlation, the enrichment of caveolin-dependent biological processes, and the convergence of experimentally validated microRNA repressors on both transcripts together describe a coherent co-expression signature rather than an incidental pattern. These data are consistent with a model in which caveolar integrity supports endothelial shear sensing, eNOS coupling, cytoskeletal stability, and calcium homeostasis, and in which its loss may contribute to a cascade of maladaptive responses that accelerate medial degeneration. The CAV1/CAV2-microRNA axis is proposed as a candidate mechanistic pathway in TAA biology and a potential target for future biomarker development and therapeutic intervention. Validation at the protein level and in mechanistic models will be required before this molecular signature can be translated into clinical tools for risk stratification or endothelial-directed therapy.

## Data Availability

The transcriptomic dataset supporting this study is openly available in the Gene Expression Omnibus (GEO) repository at https://www.ncbi.nlm.nih.gov/geo/query/acc.cgi?acc=GSE26155 (accession number GSE26155); accessed on 22 March 2025.

## Supplementary Materials

The following supporting information can be downloaded at: https://www.mdpi.com/article/10.3390/genes17070827/s1, File S1: STREGA checklist.
