# Supplementary material for: Coordinated Transcriptional Repression of CAV1 and CAV2 in Thoracic Aortic Aneurysm: A microRNA Regulatory Network Analysis
Source: Genes (Basel). 2026 Jul 20;17(7):827. doi: 10.3390/genes17070827 (PMC13411951; doi:10.3390/genes17070827)
Supplement: Supplementary file 1 [file genes-17-00827-s001.zip › Supplementary_Methods.pdf]

Supplementary Methods – Dataset Identification and Preprocessing

To investigate gene expression differences associated with thoracic aortic aneurysm (TAA), we queried the Gene Expression Omnibus (GEO) database hosted by the National Center for Biotechnology Information (NCBI) (https://www.ncbi.nlm.nih.gov/gds). This publicly accessible repository contains high-throughput gene expression data submitted by the research community. The search was performed on March 20, 2025, using a predefined query strategy. Two independent investigators (DEM, SS) systematically screened the database using the keywords: “thoracic aortic aneurysm,” “TAA,” and “aortic dilatation,” with the filter set to “Homo sapiens” to restrict results to human tissue samples. Agreement between reviewers was quantified using the kappa statistic to ensure reliability in dataset selection.

Our systematic screening identified a single microarray dataset, GSE26155, which met all predefined inclusion criteria and provided transcriptomic data appropriate for in-depth computational interrogation. This dataset encompasses 86 thoracic aortic samples, 43 aneurysmal and 43 non-aneurysmal controls, derived exclusively from human thoracic aortic tissue, including the ascending, transverse, and descending segments.

Aneurysmal specimens were collected from patients undergoing operative repair of dilated thoracic aortas with diameters exceeding 45 mm, while control samples were harvested from non-dilated donor aortas (< 40 mm) obtained during organ procurement for heart or lung transplantation. The mean aortic diameter was 53.6 ± 7.5 mm among TAA cases versus 34.1 ± 3.6 mm in controls. All subjects possessed tricuspid aortic valves, and the prevalence of aortic valve stenosis and regurgitation among aneurysmal patients was 59% and 53%, respectively. To ensure clear phenotypic separation between groups, specimens with intermediate diameters (40–45 mm) were deliberately excluded.

Transcriptomic profiling in GSE26155 was conducted using the Affymetrix Human Exon 1.0 ST Array [HuEx-1_0-st], a microarray platform capturing exon-level expression; no RNA-sequencing data were used in this study. Normalized expression values were retrieved using GEO2R (https://www.ncbi.nlm.nih.gov/geo/geo2r/), the NCBI interactive analysis tool that applies the GEOquery and limma R packages to the normalized series matrix deposited for the dataset. Expression values are background-corrected, log2-transformed, and normalized as deposited by the original investigators. For each of the five caveolae-associated genes of interest (CAV1, CAV2, CAV3, CAVIN1, CAVIN2), the platform annotation provided a single

transcript-cluster probe set per gene; the corresponding per-sample expression values were therefore used directly, and no summarization across multiple probes mapping to the same gene was required. Complete per-sample values were available for all 86 samples, with no missing values.

The extracted per-sample expression values (86 per gene) were imported into GraphPad Prism (version 10.0; GraphPad Software, San Diego, CA), in which all subsequent analyses were performed: differential expression testing (two-tailed unpaired t-tests with Welch's correction, or Mann-Whitney U tests where distributional assumptions were not met), Benjamini-Hochberg adjustment across the five candidate genes, Spearman rank correlation, Deming regression, principal component analysis (PCA), and ROC curve evaluation.
